# Acute Cardioembolic Stroke in the Setting of Subtherapeutic Anticoagulation

**DOI:** 10.7759/cureus.44925

**Published:** 2023-09-08

**Authors:** Ankit Ambatipudi, Anjali R Daniel, Rohan Mangal, Paul R Banerjee, Latha Ganti

**Affiliations:** 1 Biomedical Sciences, University of Central Florida, Orlando, USA; 2 Biology, Emory University, Atlanta, USA; 3 Medicine, University of Miami Miller School of Medicine, Miami, USA; 4 Emergency Medicine, Lakeland Regional Medical Center, Lakeland, USA; 5 Emergency Medicine, Mercer University School of Medicine, Macon, USA; 6 Medical Sciences, The Warren Alpert Medical School of Brown University, Providence, USA; 7 Emergency Medicine, University of Central Florida College of Medicine, Orlando, USA

**Keywords:** intravenous thrombolysis, middle cerebral artery infarct, subtherapeutic anticoagulation, acute cardioembolic stroke, cardioembolic strokes

## Abstract

Acute ischemic stroke is a sudden neurological deficit secondary to decreased or lack of blood flow (perfusion) due to a thrombus or an embolus. Embolic strokes are ischemic strokes that occur due to a distal clot that results in hypoperfusion upstream. Cardioembolic strokes are embolic strokes due to a cardiac origin. Almost a quarter of ischemic strokes are of cardioembolic etiology. Here, we present the case of an 83-year-old female presenting with right-side weakness and aphasia who arrived 45 minutes after symptom onset. Cardioembolic stroke symptoms, diagnosis, treatment, and risk factors are discussed.

## Introduction

Cardioembolic strokes are embolic strokes that can be traced to cardiovascular pathology, such as atrial fibrillation, left ventricular thrombi, cardiac tumors, valvular vegetations, and paradoxical emboli [1]. Cardioembolic strokes account for up to 1/4 of all ischemic strokes and are caused by various cardiovascular pathologies. The typical presentation is the occurrence of maximal neurologic deficits at onset [2]. Cardioembolic strokes typically lodge in distal arteries supplying the cerebral cortex and therefore present with cortical signs, including aphasia and visual field deficits [2]. As the population ages, the incidence of cardioembolic stroke is increasing. This is of concern, as cardioembolic strokes have the highest mortality of any stroke subtype. An emergency department cohort of consecutive patients reported a 90-day mortality rate of 36.9%, the highest of any stroke subtype [3]. This study also established cardioembolic etiology as an independent risk factor for stroke, after adjusting for both age and stroke severity. A study of 314 cardioembolic strokes in the Sagrat Cor Hospital of Barcelona Stroke Registry revealed a 77% in-hospital mortality in patients with recurrent embolic recurrence within one week [4].

## Case presentation

**Figure 1:**
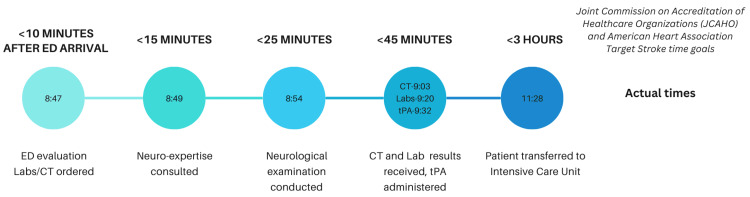
Timeline of code stroke

The patient is an 83-year-old whose family noticed right facial droop, right-side weakness, and inability to speak. She was last seen well 45 minutes prior to arrival. A code stroke was called as soon as she presented to the emergency department (ED) (Figure 1).

**Figure 2:**
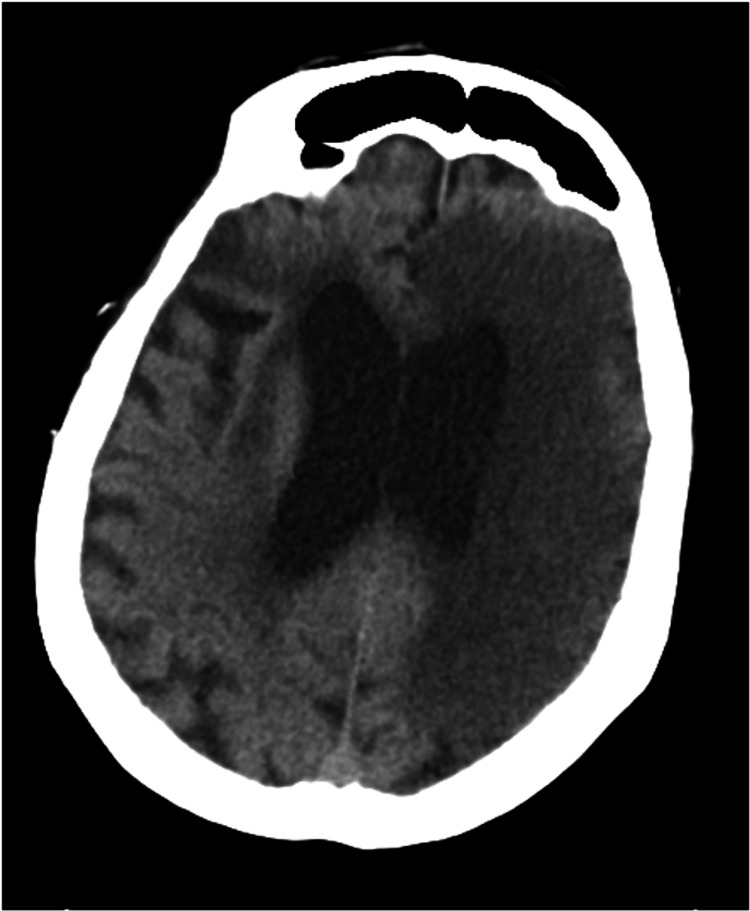
Computed tomography (CT) scan demonstrating a large hypodense area on the left, corresponding to a middle cerebral artery (MCA) infarct

The patient had a past medical history of atrial fibrillation, hypertension, congestive heart failure, and diabetes mellitus. Vital signs were temperature 97.70 F, pulse of 82 beats per minute, respiratory rate of 16 breaths per minute, blood pressure of 156/83 mmHg, and pulse oximetry of 94% on room air. Neurologic examination revealed a National Institutes of Health Stroke Scale (NIHSS) score of 26, with right-sided extremity weakness, right facial droop, left gaze deviation, right-sided neglect, and global aphasia. Brain computed tomography (CT) was negative for intracranial hemorrhage, an insular ribbon sign, a hyperdense middle cerebral artery (MCA) sign, or evidence of hypodensity. While she was within the time window for thrombolysis, the fact that she was on warfarin for her atrial fibrillation was of concern. However, laboratory analysis revealed an international normalized ratio (INR) of 1.1, suggesting that she was subtherapeutic. The family then confirmed that she had not been taking her medications for a few days. Given the lack of contraindications, the patient was thrombolysed with alteplase and admitted to the intensive care unit. Although the patient's NIHSS decreased to 25 (mildly improved extremity weakness), repeat CT 24 hours after admission revealed a large infarct in the left MCA territory (Figure 2).

## Discussion

This case illustrates a large MCA territory infarct in a patient with several cardiovascular risk factors. Her CHA_2_DS_2_-VASc Score for Atrial Fibrillation Stroke Risk adds up to 6, with a point each for being female and having hypertension, congestive heart failure, and diabetes, plus two points for being over the age of 75. A score of 6 is associated with a stroke risk of 9.7% per year in >90,000 patients in the Swedish Atrial Fibrillation Cohort Study [5]. Strokes are classified according to the trial of ORG 10172 in acute stroke treatment (TOAST) classification [6], which identifies categories: large artery disease, cardioembolism, small vessel occlusion, other determined etiology, and undetermined etiology. Diagnosis is based on data from laboratory evaluation and results of brain imaging. There are various etiologies for cardioembolic stroke (Table 1).

**Table 1:** Etiologies of cardioembolic stroke

Causes of cardioembolic stroke
Recent myocardial infarction
Mechanical prosthetic valve
Dilated myocardiopathy
Mitral rheumatic stenosis
Prosthetic valve
Significant mitral stenosis
Left atrial or ventricular thrombus
Dilated cardiomyopathy
Akinetic/hypokinetic left ventricular segment
Atrial myxoma
Infective endocarditis
Sick sinus syndrome
Congestive heart failure
Rheumatic heart disease
Patent foramen ovale with thrombus in situ

The treatment and management for cardioembolic strokes involve the use of anticoagulants for secondary prevention. The goal of the workup of cardioembolic stroke is to detect the presence of a potential intracardiac source of the embolism. Our patient had atrial fibrillation, an arrhythmia that increases the risk of stroke by fivefold, and one of the most common causes of cardioembolic strokes [1]. Warfarin has been shown to mitigate the risk of stroke in such patients and is superior to aspirin [7]; thus, our patient was on the appropriate therapy. Warfarin is generally used for oral anticoagulation with a target INR of 2.0 to 3.0. However, her subtherapeutic INR, likely secondary to medication non-adherence, put her at increased risk. A study of 626 patients highlights the underuse of oral anticoagulation as a risk factor for stroke associated with atrial fibrillation [8]. Subtherapeutic anticoagulation of patients with cardioembolic stroke has been attributed to clinicians’ fears of putting elderly patients on warfarin due to the risk of intracranial hemorrhage and falls [9]. However, the burden of cardioembolic stroke is significant, so anticoagulation is of paramount importance. With the advent of direct oral anticoagulants, patients and clinicians have more choices for anticoagulation. Furthermore, there is now a United State Food and Drug Administration (US FDA) approved antidote of the factor Xa inhibitors, andexanet alfa, which may further mitigate the hesitancy of putting older patients on direct oral anticoagulants.

## Conclusions

Atrial fibrillation confers a significant burden of cardioembolic stroke and is seen more commonly due to the aging population. The use of anticoagulants is the mainstay of treatment for atrial fibrillation, and discontinuing anticoagulation results in subtherapeutic anticoagulation. This in turn increases the risk of cardioembolic events, which can result in devastating strokes.
